# Supplementary material for: Designed Ankyrin Repeat Proteins provide insights into the structure and function of CagI and are potent inhibitors of CagA translocation by the Helicobacter pylori type IV secretion system
Source: PLoS Pathog. 2023 May 8;19(5):e1011368. doi: 10.1371/journal.ppat.1011368 (PMC10194873; doi:10.1371/journal.ppat.1011368)
Supplement: S1 Table — (DOCX) [file ppat.1011368.s001.docx]

**S1 Table. Small angle X-ray scattering data collection, processing and analysis**

|  | CagI | CagI^N^ | CagI^C^ |
| --- | --- | --- | --- |
| **Data-collection parameters:** |  |  |  |
| Q range (Å^-1^) | 0.01-0.17 | 0.01-0.18 | 0.01-0.23 |
| **Structural parameters:** |  |  |  |
| I(0) (Å^-1^) (from P(r)) | 18.2 | 5.7 | 9.4 |
| Rg (Å) (from P(r)) | 4.66 ± 0.03 | 2.9 ± 0.1 | 2.4 ± 0.02 |
| I(0) (Å^-1^) (from Guinier) | 18.2 | 5.7 | 9.4 |
| Rg (Å) (from Guinier) | 4.65 ± 0.07 | 2.9 ± 0.1 | 2.3 ± 0.02 |
| Dmax (Å) | 162 | 116 | 88 |
| Porod volume estimation. V_0_ (Å^3^) | 155.3 | 54.4 | 27.8 |
| **Molecular-mass determination:** |  |  |  |
| Molecular mass Mr (kDa) (SaxsMOW) | 99.1 | 32 | 20.3 |
| Molecular mass Mr (kDa) (Porod) | 91.3 | 32 | 16.3 |
| Calculated mass Mr (kDa) (sequence) | 78.2 | 35.6 | 18.1 |
