# Supplementary figures and images for: Designed Ankyrin Repeat Proteins provide insights into the structure and function of CagI and are potent inhibitors of CagA translocation by the Helicobacter pylori type IV secretion system
Source: PLoS Pathog. 2023 May 8;19(5):e1011368. doi: 10.1371/journal.ppat.1011368 (PMC10194873; doi:10.1371/journal.ppat.1011368)

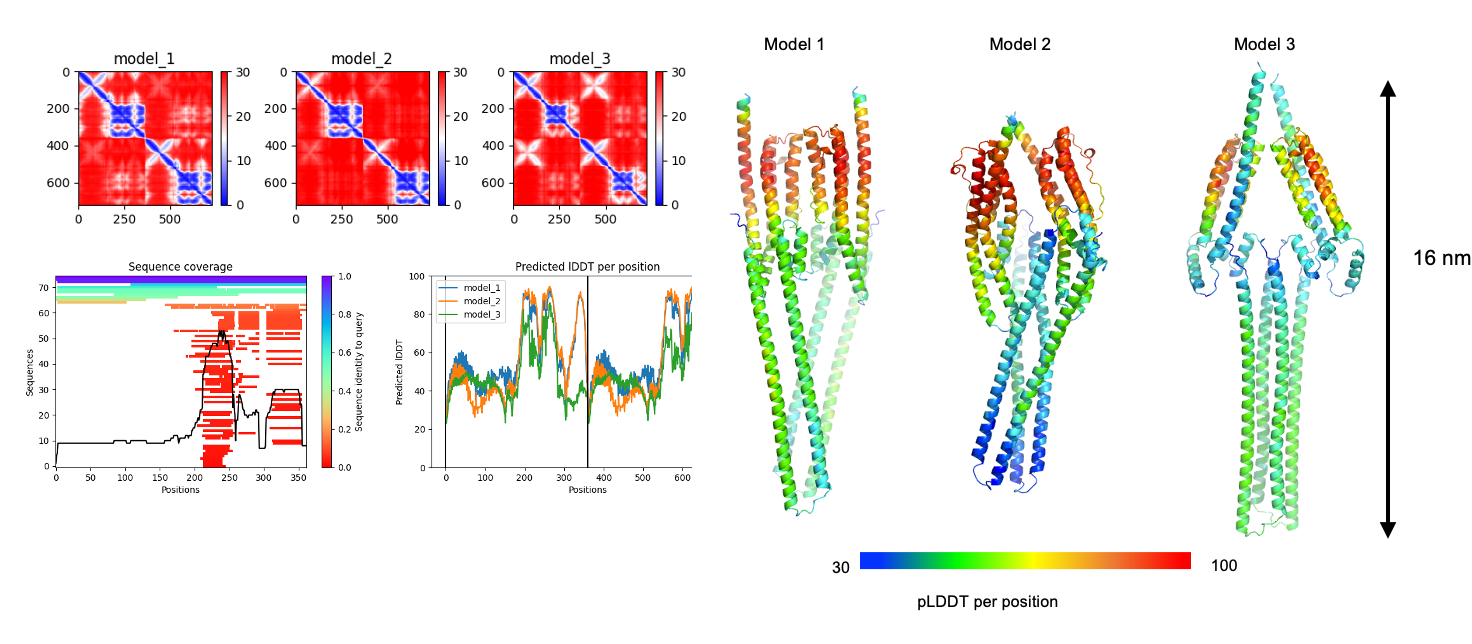

Supplement: S1 Fig — Scores and sequence coverage of the AF models (left) and cartoon depiction of the AF models of CagI dimer (right) coloured according to pLDDT scores (30 to 100). (TIF) [file ppat.1011368.s002.tif]

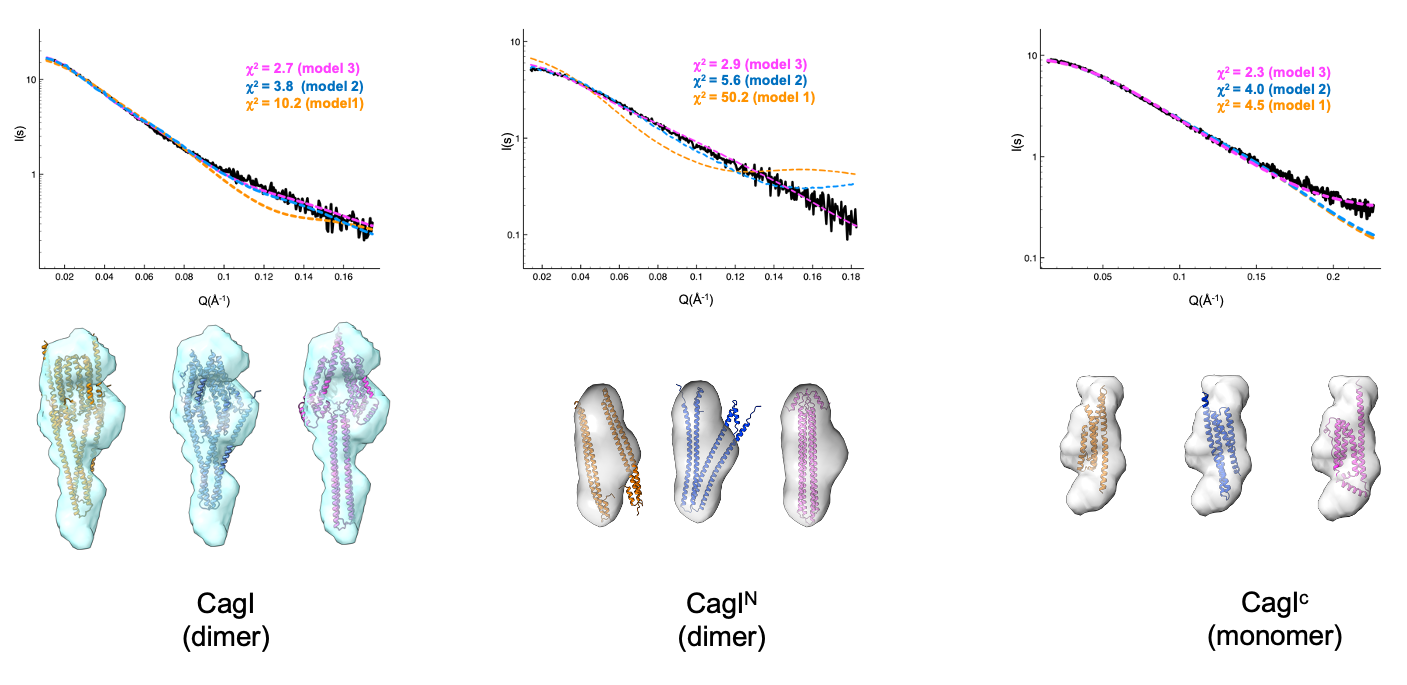

Supplement: S2 Fig — Size exclusion coupled to experimental SAXS curves of CagI (black), CagIN and CagIC compared to theoretical curves obtained with the corresponding model 1, 2 and 3 and domains. Theoretical curves were obtained with CagI dimers, CagIN dimers and CagIC monomers (chain A). Below are cartoon representations of the structures of model 1 (orange), model 2 (blue) and model 3 (magenta) fitted into the DAMMIN envelope obtained for each SAXS data. From left to right: full length CagI dimer, CagIN dimer and CagIC monomer. (TIF) [file ppat.1011368.s003.tif]

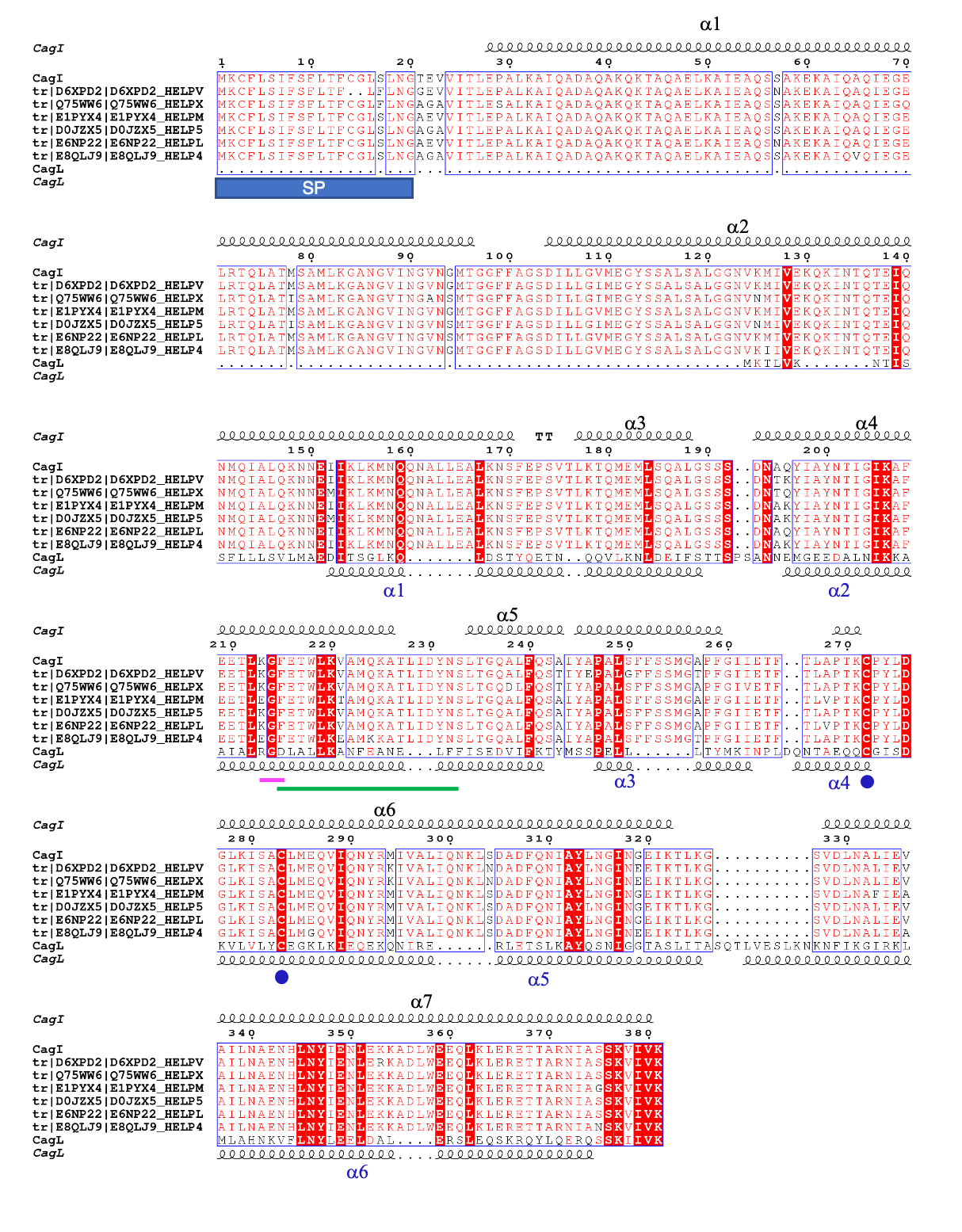

Supplement: S3 Fig — Clustal O alignment of CagI sequences. CagI sequence from strain 26695 (Uniprot O25273) is labelled CagI. The other CagI sequences are labelled with their uniprot code and have been chosen to illustrate the low diversity. CagL sequence corresponds to the one from strain 26695 (Uniprot O25272). Secondary structures of CagI (AF model) and CagL (PDB code 3ZCJ) are indicated above and below the alignment, respectively. The predicted signal peptide sequence (SP) is indicated by a blue box, the cysteines forming disulfide bridges are indicated by a blue dot. The arginine-glycine-aspartate [21] and D1 motifs [44] of CagL are indicated by magenta and green lines, respectively. (TIF) [file ppat.1011368.s004.tif]

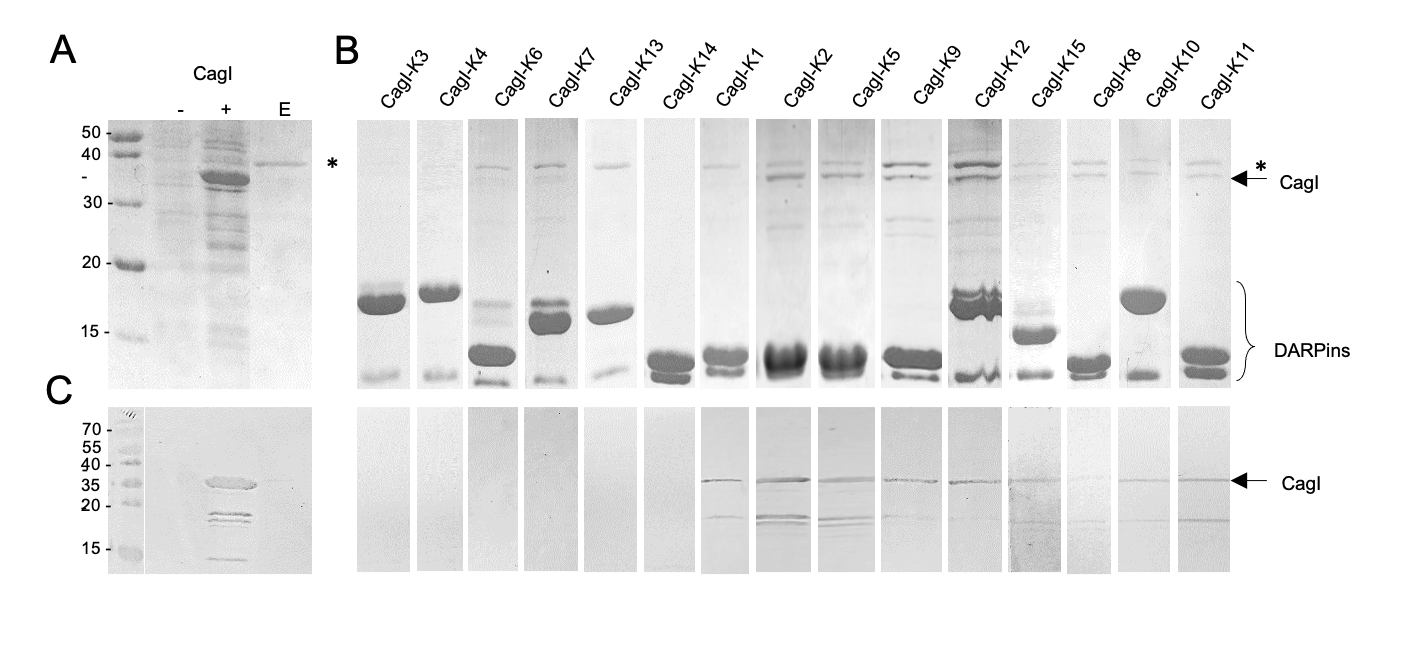

Supplement: S4 Fig — Coomassie blue stained SDS-PAGE of A) E. coli cell extracts before (-), after (+) induction of CagIstrep expression and (E) elution fraction of His-trap column. No CagI protein could be detected in this fraction but a contaminant of around 39 kDa is visible and indicated by a *. B) co-purification of His8-DARPins (K1-K15) with CagI-Strep on Ni-NTA beads showing that a band corresponding to CagIstrep is present in the elution fraction when co-expressed with K2, K5, K9, K12, K8, K10, K11 and K15 but not with K3, K4, K6, K7, K13, K14 and K1. C) Western-blot analysis of the same samples using anti-strep antibody and stained with NBT-BCIP. The band corresponding to CagIstrep is indicated by an arrow. (TIF) [file ppat.1011368.s005.tif]

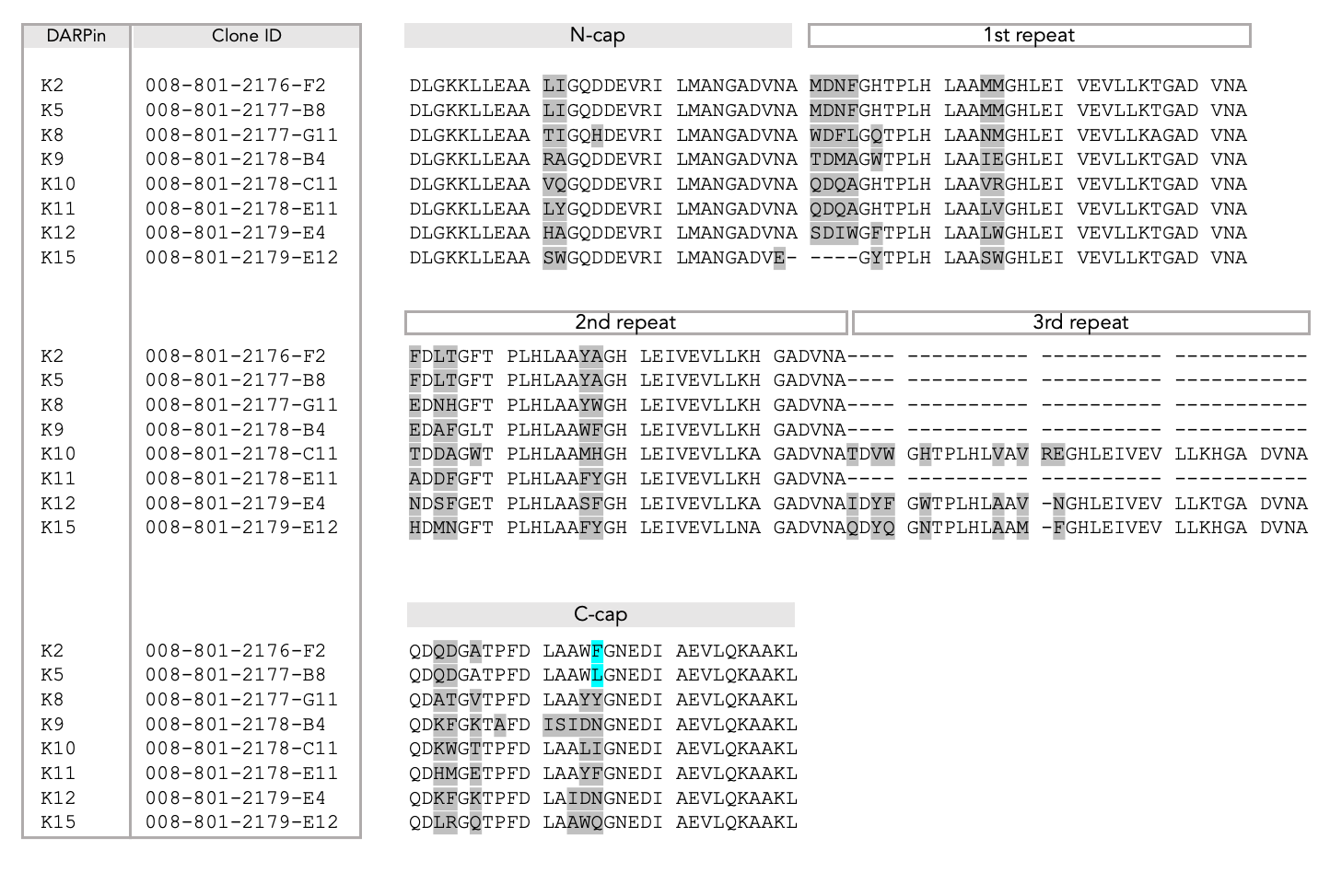

Supplement: S5 Fig — Sequences of the DARPins, with name and clone ID indicated, aligned using ClustalO. Amino-acid differences are shaded in grey except for the single difference between K2 and K5 shaded in cyan. Repeat, N-cap and C-cap regions are indicated above the sequences. (TIF) [file ppat.1011368.s006.tif]

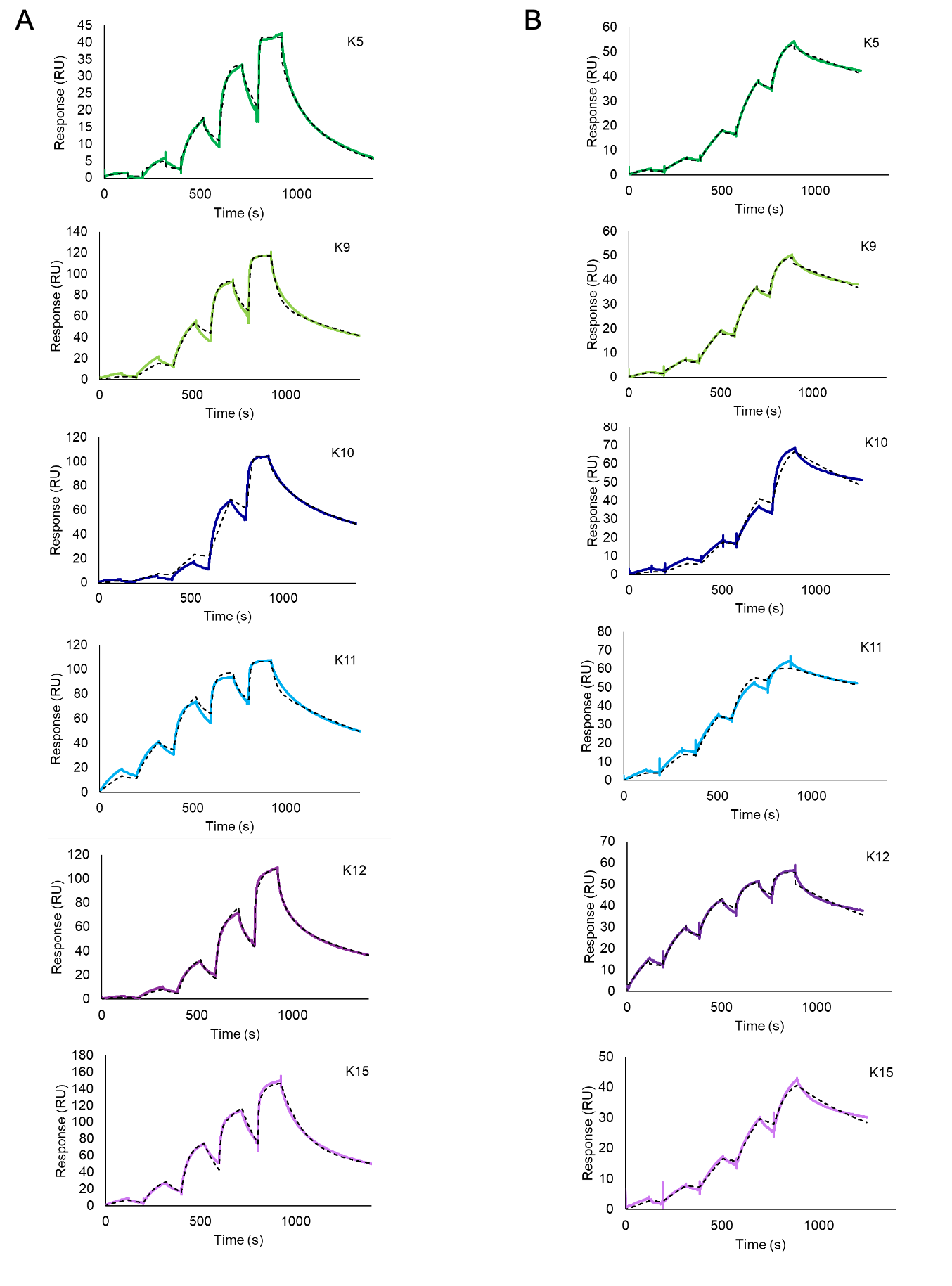

Supplement: S6 Fig — Surface Plasmon Resonance experiments using single-cycle-mode on CM5 chips coated with CagI (A) or with (B) CagIC. DARPins were injected on the chips at increasing concentrations as follows. For experiments performed on full-length CagI, concentrations of DARPins were: 0.5, 2.5, 12.5, 62.5 and 312.5 nM for K9, K11, K12, K15; and 1, 3, 9, 27 and 81 nM for K10. For CagIC experiments, injections of DARPins were performed with concentrations of 0.05, 0.15, 0.45, 1.35 and 4 nM for K9, K10 and K11; 0.16, 0.8, 4, 20 and 100 nM for K12; and 2, 4, 8, 16 and 32 nM for K15. Fit curves obtained with heterogenous ligand model (CagI) or binding model 1:1 (CagIC) are shown as black dashed lines. (TIF) [file ppat.1011368.s007.tif]

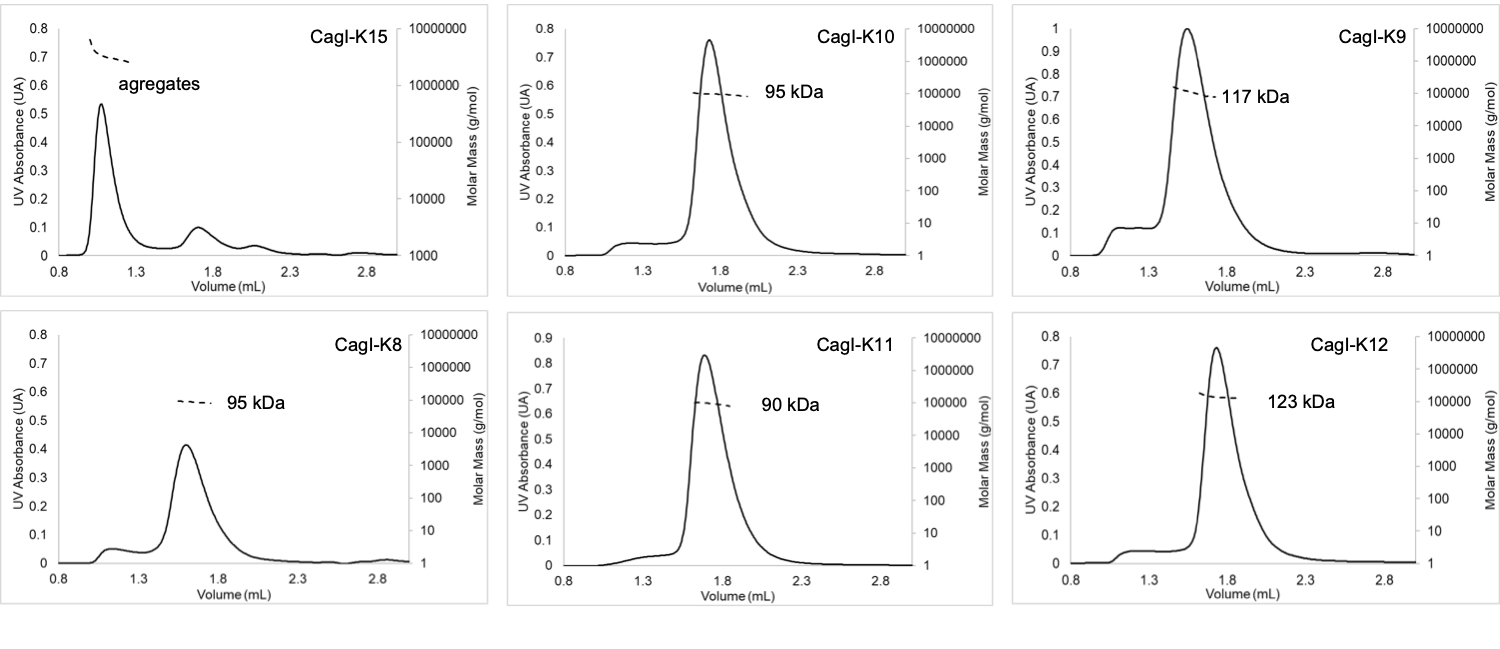

Supplement: S7 Fig — Each purified CagI:DARPin complex was submitted to SEC-MALS measurement represented by A280 chromatograms. Molar mass calculations are represented by dotted lines on each graph. (TIF) [file ppat.1011368.s008.tif]

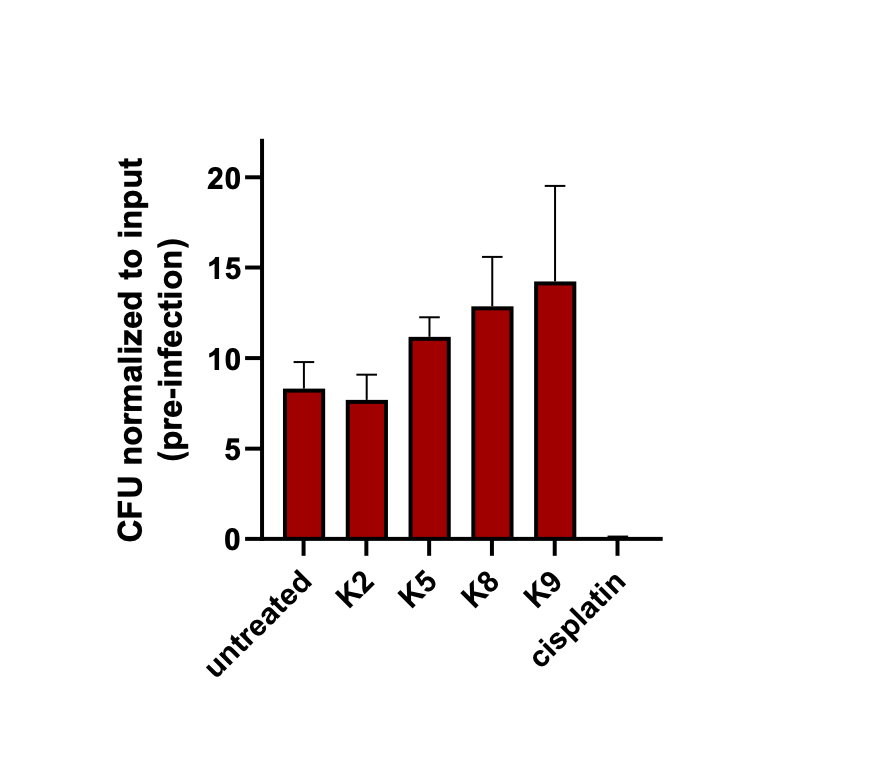

Supplement: S8 Fig — H. pylori strain P12 was treated with DARPins or cisplatin in PBS/10% FCS for 30 min at 37°C, or left untreated, as indicated, and co-incubated with AGS cells for 2.5 h at 37°C, 5% CO2, Subsequently, cells were washed to remove unbound bacteria and detached by EDTA treatment, and colony-forming units (CFU) were determined by plating serial dilutions on serum agar plates. Data are indicated as normalized values (percentages in relation to CFU of untreated bacteria prior to co-incubation), and represent mean values and standard errors of the mean, resulting from three to four independent experiments. (TIF) [file ppat.1011368.s009.tif]

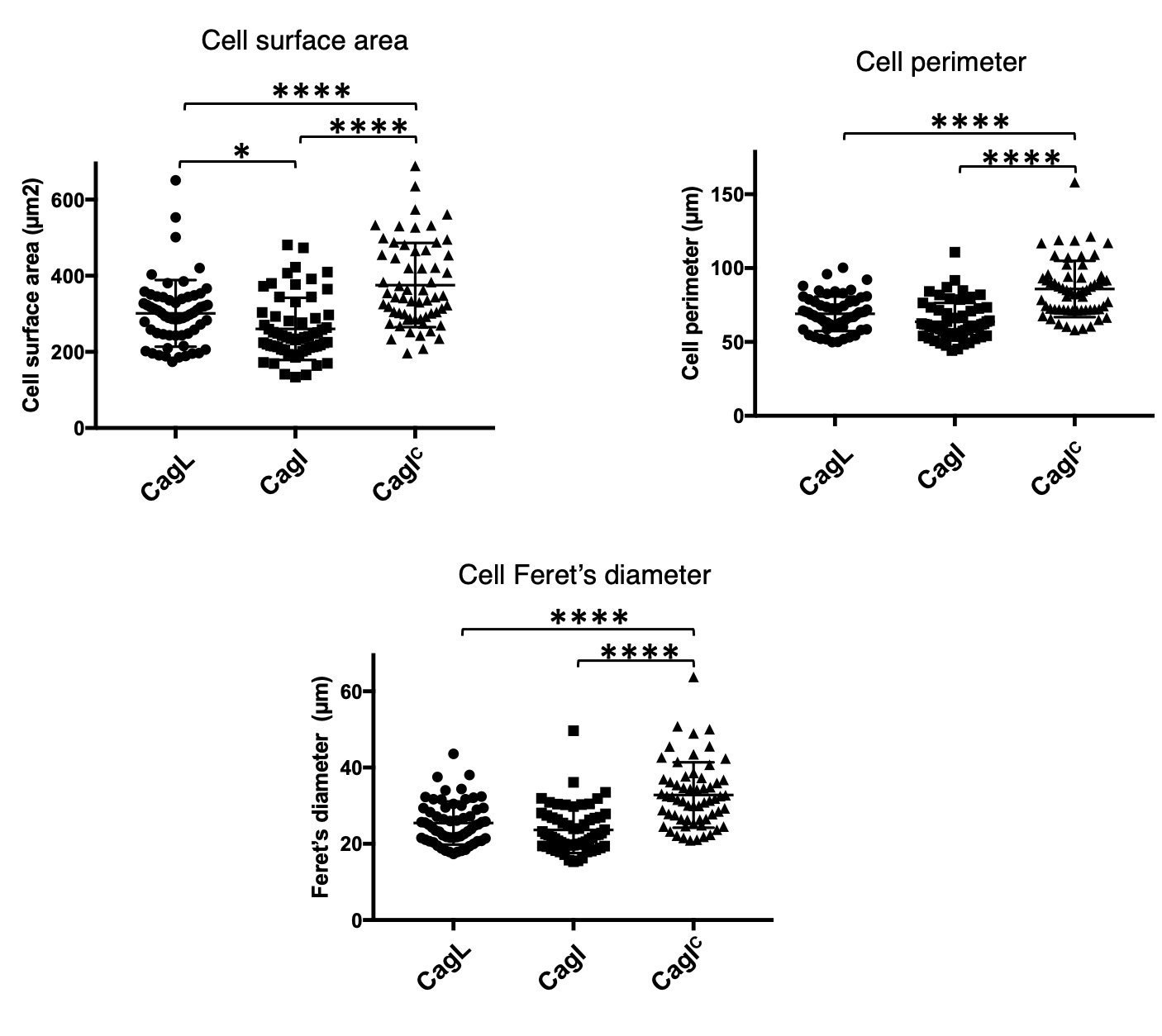

Supplement: S9 Fig — Morphological characterization of cells adhered to 0.15 μg of CagL, CagI, CagIC 60 minutes after seeding. Cell surface area, perimeter and Feret’s diameter were determined on phase contrast images using Fiji software. Each dot represents one cell (60 adhered AGS cells were measured for each condition). Each of the three parameters clearly shows that cells that have adhered to CaglC have a larger and more extended contact surface compared to cells bound to CagI or CagL. A one-way ANOVA with Tukey’s post-test was used to determine significance. Means ± SD are shown with *p<0.1, ****p<0.0001. (TIF) [file ppat.1011368.s010.tif]

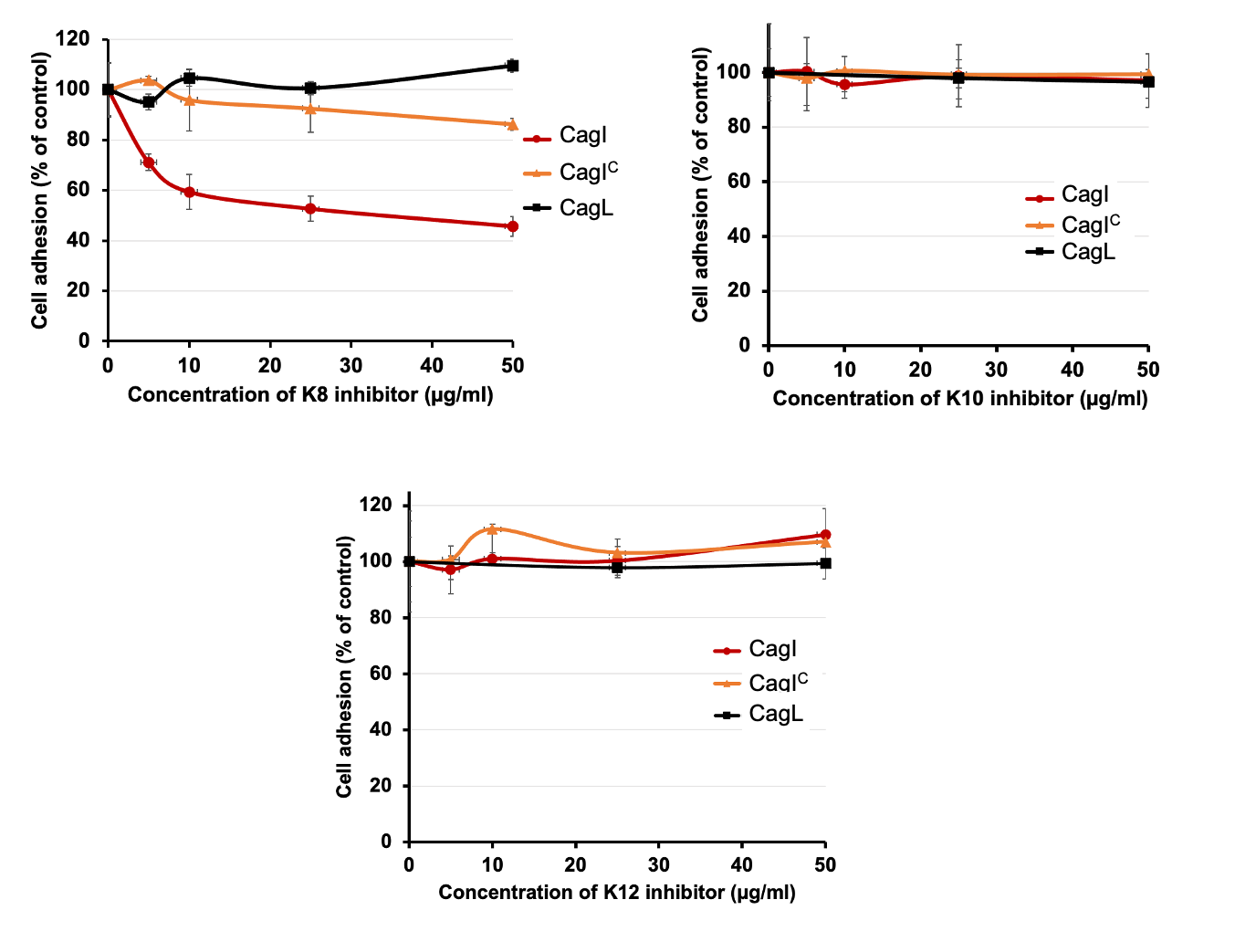

Supplement: S10 Fig — Effect of DARPin K8, K10 or K11 on adhesion of AGS cells to CagL, CagI, CagIC. Multiwell plates were coated with 4 μg of each protein. After saturation with 1% BSA, the wells were incubated with 50 μL of the indicated concentration of DARPin for 1 h at room temperature, and the cells were seeded in the presence of the inhibitor. The extent of adhesion was measured as previously described and expressed as percentage of adhesion to each protein in the absence of the inhibitor. Each assay point was derived from triplicate measurements. (TIF) [file ppat.1011368.s011.tif]
